# Osteoporosis in Systemic Mastocytosis: A Scoping Review

**DOI:** 10.3390/medicina60111752

**Published:** 2024-10-24

**Authors:** Giulia Letizia Mauro, Jessica Accomando, Sofia Tomasello, Adele Duca, Maria Silvia Mangano, Alessandro de Sire, Michele Vecchio, Dalila Scaturro

**Affiliations:** 1 Precision Medicine in the Medical, Surgical and Critical Care Areas, University of Palermo, 90100 Palermo, Italy; giulia.letiziamauro@unipa.it; 2 Section of Pharmacology, Department of Biomedical and Biotechnological Sciences, University of Catania, 37100 Catania, Italy; jessica.accomando9@gmail.com (J.A.); adeleduca93@gmail.com (A.D.); mariasilvia.mangano@gmail.com (M.S.M.); michele.vecchio@unict.it (M.V.); 3 Neuromotor and Cognitive Rehabilitation Research Center, Physical and Rehabilitation Medicine Section, Department of Neurosciences, Biomedicine and Movement Sciences, University of Verona, 37100 Verona, Italy; sofia.tomasello@gmail.com; 4 Physical and Rehabilitative Medicine, Department of Medical and Surgical Sciences, University of Catanzaro “Magna Graecia”, 88100 Catanzaro, Italy; alessandro.desire@unicz.it; 5 Rehabilitation Unit, AOU Policlinico Vittorio Emanuele, 95123 Catania, Italy

**Keywords:** systemic mastocytosis, secondary osteoporosis, BMD, osteoporotic fractures, bone densitometry, radiography, vertebral morphometry, bone scintigraphy, bisphosphonate drug therapy, denosumab

## Abstract

*Background*: Mastocytosis (MS) is a rare disease that can involve various organs, including the bone. Given the incidence of the disease in the global population, MS poses a challenge for physicians, and early therapeutic intervention in the initial stages could significantly impact the quality of life of affected patients. *Objective*: The aim of this scoping review was to provide an overview of secondary osteoporosis in systemic mastocytosis (SM), focusing on the heterogeneity of its manifestations, the benefits of early diagnosis, and appropriate pharmacological treatment. *Design*: A technical expert panel (TEP) consisting of 8 physicians with expertise in metabolic bone diseases conducted the review following the PRISMA-ScR model. A strength of this study is that it provides various therapeutic approaches for patients with bone involvement in SM, although the limited available literature on the topic constituted a limitation. The TEP sought evidence regarding the following diagnostic and therapeutic modalities in the management of SM: “bisphosphonate therapy”, “zoledronic acid therapy”, “denosumab therapy”, “IFN-alpha therapy”, and “IFN-alpha therapy in combination with pamidronate”. *Results*: Clinical data showed a correlation between densitometric outcomes, serum tryptase levels, and mast cell infiltration in the bone marrow, between increased bone mineral density and the presence of osteosclerosis in cases of advanced SM, between the severity of osteoporosis and hypertryptasemia, and also provided results on the long-term effects of bisphosphonate therapy, the therapeutic efficacy of zoledronic acid administration, the positive effect of denosumab on the reduction of serum tryptase levels (even if is proved in a limited numbers of cases) and the prevention of new fractures, and the effect of IFN-alpha in more severe cases of SM, either alone or in combination with pamidronate. *Conclusions*: Studies have demonstrated the effectiveness of various treatments depending on the form of mastocytosis, whether indolent systemic or advanced systemic, in the prognosis of the disease. However, this role should be further investigated in additional clinical studies, considering the limited data on the use of these interventions.

## 1. Introduction

Mastocytosis is a rare disease characterized by abnormal proliferation of mast cells and various manifestations. In 80% of cases, the disease is caused by the generally acquired mutation of the Kit gene D816V (Kit Proto-Oncogene, Receptor Tyrosine Kinase), which encodes a transmembrane receptor with tyrosine kinase activity, but it can also be caused by other mutations of the same gene. These mutations lead to persistent activation of the c-kit surface receptor, which is usually only responsive to stem cell factor (SCF), playing an important role in the regulation of proliferation, maturation, adhesion, chemotaxis, and survival of mast cells. Mast cells with the KIT D816V mutation thus exhibit an increased proliferative index, a tendency to aggregate into atypical spindle-shaped mast cell clusters, and an increased expression of lymphocytic markers such as CD2 and CD25 [1].

The World Health Organization classifies mastocytosis into two main forms: cutaneous and systemic [2]. Cutaneous mastocytosis, more common in childhood, usually has a self-resolving pattern with few cases persisting into adulthood and may present with urticaria, itching, erythema, and angioedema. In contrast, the onset in adulthood usually manifests as a systemic form. SM is a chronic disease defined by involvement of at least one extracutaneous organ, the most common of which are the spleen, lymph nodes, bone marrow, bones, and gastrointestinal tract. Generally, the course of SM can be indolent (ISM) and patients are typically managed with symptomatic therapies; rarely, the disease can progress to more severe forms with an unfavorable prognosis, such as aggressive systemic mastocytosis (ASM), systemic mastocytosis with associated non-mast cell hematologic disease (SM-AHN), mast cell sarcoma (MC), and mast cell leukemia (MCL). There is also an intermediate or “smoldering” mastocytosis (SSM), without organ dysfunction and with a benign prognosis [3,4].

The disease is primarily due to the release of mediators by mast cells, such as histamine, heparin, leukotrienes, and various inflammatory cytokines, and can be triggered by contact, physical exertion, alcohol, NSAIDs (non-steroidal anti-inflammatory drugs), opioids, insect stings, or food intake. The systemic release of these mediators can cause gastric symptoms (diarrhea, nausea, vomiting), loss of bone mass, and anaphylaxis. In its most severe form, tissue invasion can lead to hypersplenism, liver disease, ascites, malabsorption, focal bone lesions, and cytopenia [5].

Bone involvement in SM is very common and often asymptomatic. Osteoporosis and bone fragility affect about 20 percent of cases. Bone damage, which may be directly induced by mast cell proliferation, is heterogeneous, with both osteolytic and osteosclerotic patterns occurring simultaneously, complicating diagnosis and therapeutic approaches [6].

Various therapeutic approaches may be administered to counteract secondary osteoporosis, including bisphosphonates, zoledronic acid, biological therapy, and denosumab, which have shown adequate compliance in complex patients [7].

There is still no consensus on specific anti-osteoporotic treatment in patients with systemic mastocytosis [8].

In this context, with this scoping review, we aimed to provide an overview of secondary osteoporosis in systemic mastocytosis in order to define early and appropriate pharmacological treatment [9].

## 2. Materials and Methods

This review was conducted according to the PRISMA-ScR model (Preferred Reporting Items for Systematic Reviews and Meta-Analyses Extension for Scoping Reviews). The technical expert panel (TEP) consisted of eight physicians, including two physiatrists specializing in metabolic bone diseases (GLM, DS), two experts in scoping review methodology (ADS, MV), and four residents (JA, AD, MSM, SF). It was not necessary to submit this manuscript to an ethics committee or register a clinical study, as this is a systematic review.

The TEP examined the biological effects and clinical efficacy of the following pharmacological therapies for osteoporosis secondary to mastocytosis: bisphosphonate therapy, zoledronic acid therapy, denosumab therapy, IFN-alpha therapy, and IFN-alpha combined with pamidronate therapy.

### 2.1. Search Strategy

The TEP performed the search on PubMed (Public MedLine, maintained by the National Center of Biotechnology Information (NCBI) of the National Library of Medicine, Bethesda, MD, USA), with a search string combining keywords for both mastocytosis and osteoporosis (see Table 1 for further details on the search strategy).

### 2.2. Selection of Studies

The TEP considered articles published from 1996 to 31 December 2023, for eligibility, including those in English and French (see Table 2 for further details on the eligibility criteria). All data extracted from full texts and the results of the included studies were analyzed qualitatively.

## 3. Results

**Figure 1:**
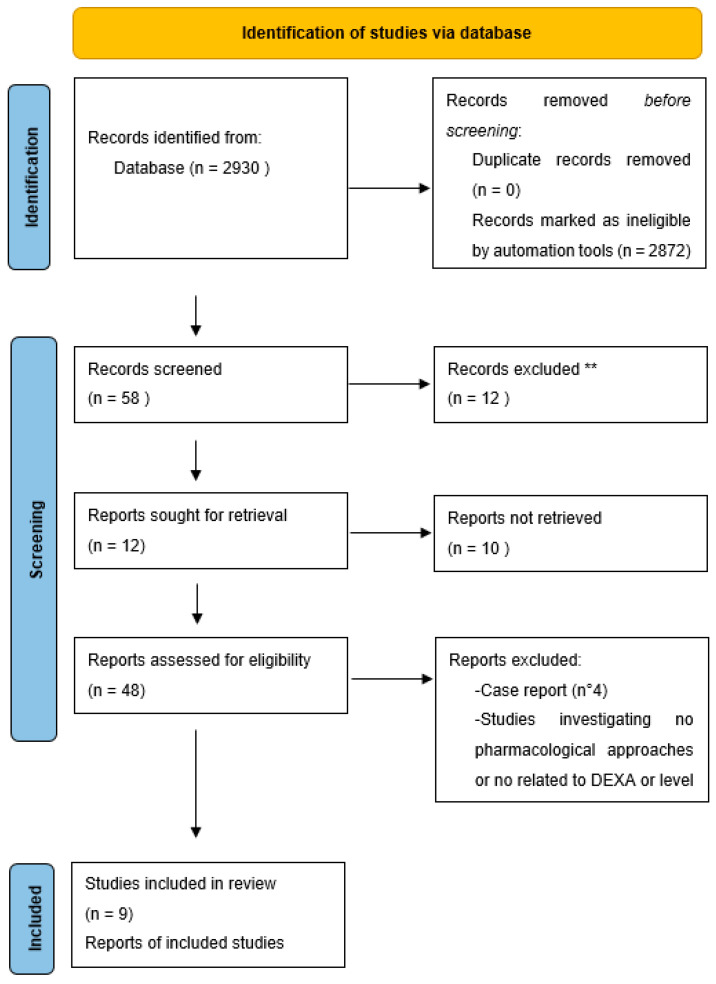
PRISMA-ScR flow diagram of the study selection process. ** Legend. Initially, the TEP found 2930 articles in the PubMed database. Based on the titles and abstracts, and following our exclusion criteria, a total of 2886 articles were excluded. Additionally, 39 articles were excluded after reading the full text because they did not meet our inclusion criteria (total exclusions: 2921). The remaining nine articles (published between January 1996 and December 2021) met the inclusion criteria.

Initially, the TEP found 2930 articles in the PubMed database. Based on titles and abstracts, and following our exclusion criteria, a total of 2886 articles were excluded. Additionally, 39 articles were excluded after reading the full text because they did not meet our inclusion criteria. The remaining nine articles (published between January 1996 and December 2021) met the inclusion criteria (Figure 1). All studies included in our analysis were observational clinical studies. Three of these focused on correlations between densitometric values, trypsin levels, and bone marrow infiltration of mast cells in patients with systemic mastocytosis, thus exploring the potential for early diagnosis and prognosis. The other six evaluated different pharmacological treatments in patients with various forms of mastocytosis. Among these studies, the TEP identified one study assessing the long-term effects of bisphosphonate therapy, one study on the effectiveness of zoledronic acid in reducing bone turnover markers, one study on denosumab and the risk of new fractures, one study on the effects of IFN-alpha administration, one study on the administration of pamidronate after IFN-alpha to reduce side effects, and one final study on the combined administration of the two molecules (see Table 3 for further details on the included studies).

## 4. Aetiopathogenesis in Bone Involvement

Osteoporosis is a prevalent complication in systemic mastocytosis, primarily affecting the vertebrae and flat trabecular bones, often resulting in fragility fractures. A minority of patients may present with single or multiple lytic or sclerotic lesions, reduced bone mineral density (BMD), and elevated bone turnover, accompanied by a diffuse absorption pattern on bone scintigraphy. Therefore, symptoms such as unexplained fragility fractures and low BMD in patients with SM should raise suspicion for secondary osteoporosis. The pathophysiology of skeletal changes in SM is complex and not well understood. Elevated concentrations of mast cells have been associated with increased bone turnover, while their deficiency is linked to delayed osteoclastic recruitment and osteoblastic formation. It is believed that varying degrees of tissue infiltration by neoplastic mast cells and the release of mast cell mediators are responsible for the different skeletal manifestations in SM [19]. Histamine is a key mediator in this process, stimulating osteoclasts and their precursors through autocrine and paracrine pathways [20].

Experimental studies, such as those by C. Dobigny and JL Saffar, have demonstrated the role of histamine in bone resorption. Administration of antihistamines like mepyramine and cimetidine, which bind respectively to H1 and H2 receptors on osteoclasts, resulted in reduced osteoclastic activity and bone resorption [21]. Additionally, studies on histidine decarboxylase knockout mice, an enzyme necessary for histamine production, showed a reduced number of osteoclasts and increased bone formation, indicating a protective effect against osteoporosis [22].

Further research by Martin Biosse-Duplan et al. highlighted that osteoclast precursors are the main source of histamine. This histamine directly influences osteoclasts and indirectly increases the expression of receptor activator of nuclear factor kappa-B ligand (RANKL) by osteoblasts. These findings suggest a potential protective effect of H1 and H2 receptor antagonists, concluding that antihistamine compounds or histidine decarboxylase inhibitors may be used to inhibit osteoclastic activity and bone resorption in secondary osteoporosis related to systemic mastocytosis [23].

RANKL, produced by osteoblasts and other cells such as bone marrow stromal cells, along with osteoprotegerin (OPG), a decoy receptor for RANKL, are essential in bone remodeling. Elevated levels of both RANKL and OPG have been reported in patients with mastocytosis, indicating the involvement of the RANKL/RANK/OPG pathway in mastocytosis-related osteoporosis. Mast cells themselves can produce RANKL and OPG [24,25]. Cytokines such as TGF-beta, FGF, and VEGF, synthesized by mast cells, contribute to tissue remodeling, causing liver and bone marrow fibrosis and increasing bone turnover [20]. Biochemical markers of bone turnover (BTM), such as bone-specific alkaline phosphatase (bALP), collagen type 1 telopeptide (CTX), and serum osteocalcin, help quantify systemic bone remodeling activity. However, studies have shown that BTMs are not always predictive of vertebral fractures in patients with indolent systemic mastocytosis, as they can be normal, elevated, or even below normal ranges [26,27].

Given the limitations of assessing serum BTMs, they cannot reflect focal bone lesions or the local activity and balance of multicellular bone units. Instead, they indicate only a generalized increase in bone turnover and remodeling. When BTMs fall within the normal range, this suggests a generalized increase in bone turnover, but does not exclude a local imbalance between the activity of osteoblasts and osteoclasts. Another explanation for fractures in patients with normal serum levels of BTMs could be local exposure to mast cell mediators without systemic effects. These findings imply that osteoporosis related to mastocytosis might be characterized not only by an absolute increase in osteoclastic activity but also by a relative or localized predominance of osteoclastic activity over osteoblastic activity [26,28].

Elevated serum levels of CTX have been independent predictors of future fragility fractures in patients with indolent systemic mastocytosis, correlated with serum tryptase levels, which are also correlated with levels of bALP. This suggests a link between the bone remodeling process and the number of mast cells, highlighting the complexity of bone turnover in mastocytosis [28,29]. In their evaluation of patients with ISM, M. Rossini et al. observed a significant correlation between serum tryptase levels and serum levels of bALP, a marker of bone formation [30]. They also found that very high serum tryptase levels were associated with diffuse osteosclerosis [26]. These correlations between serum tryptase levels and the BTM, as well as between BTMs and disease aggressiveness, indicate a connection between the bone remodeling process and the number of mast cells.

The differentiation of osteoblasts is primarily regulated by the canonical wingless-related integration site (WNT) pathway, which acts as the main regulator of osteogenesis alongside bone morphogenetic proteins. This pathway is crucial in determining the fate of mesenchymal stem cells. Without β-catenin, these cells do not differentiate into mature osteoblasts expressing osteocalcin but instead become chondrocytes. The WNT pathway also promotes osteoblastogenesis by inhibiting adipogenesis through the suppression of PPARγ (peroxisome proliferator-activated receptor gamma). Under certain conditions, reduced WNT signaling can lead to increased osteoclastogenesis and bone resorption by decreasing osteoblastic expression of OPG. The WNT pathway in bone is primarily regulated by receptor inhibitors such as DKK1 and sclerostin. Sclerostin regulates the late differentiation of osteoblasts and pre-osteocytes, while the expression of DKK1 is limited to osteoblasts and mature osteocytes. Elevated levels of sclerostin or DKK1 are associated with osteopenia or osteolytic lesions [31]. Recent, albeit inconsistent, reports have indicated increased levels of sclerostin or DKK1 in patients with indolent systemic mastocytosis. [24,32]. These findings suggest that the WNT/β-catenin pathway may also be disrupted in patients with systemic mastocytosis, leading to inadequate bone formation and contributing to low bone mass. Furthermore, serum levels of sclerostin and 25-hydroxyvitamin D have been found to be negatively correlated, indicating that vitamin D levels may play a crucial role in determining sclerostin levels in patients with systemic mastocytosis [33].

## 5. Diagnosis

According to the World Health Organization (WHO), the diagnosis of systemic mastocytosis requires specific criteria. One major criterion involves the detection of aggregates of at least 15 mast cells in biopsy samples from the bone marrow or other extracutaneous organs. Additionally, there are four minor criteria: the presence of atypical mast cells (>25%) on the peripheral smear, an aberrant immunophenotype with abnormal expression of non-mast cell markers (CD25 and/or CD2), a point mutation in codon 816 of the KIT gene in the bone marrow, peripheral blood, or other extracutaneous sites, and elevated serum tryptase levels (greater than 20 ng/mL). For a diagnosis, at least one major criterion and one minor criterion, or three minor criteria, must be met [34]. Diagnosing SM in the absence of skin involvement, particularly in patients with indolent systemic mastocytosis and a low mast cell burden, such as isolated bone marrow mastocytosis (BMM), is challenging. In these cases, serum tryptase levels are often normal or near-normal. Therefore, SM should be suspected in cases of unexplained recurrent anaphylaxis, flushing, osteoporosis, gastrointestinal ulcer disease, or chronic abdominal cramps [35].

Mast cell proliferation in the bone marrow is common in SM and is often correlated with radiographically detectable bone lesions. However, the pathological correlates of skeletal anomalies are poorly characterized. Histomorphometric analysis of trabecular bone in SM reveals accelerated bone remodeling characterized by osteoid accumulation, peritrabecular fibrosis, increased numbers of osteoblasts and osteoclasts, and increased osteoclastic resorption surfaces [36]. The diagnosis of osteoporosis secondary to mastocytosis requires a combination of laboratory and instrumental examinations. Radiological findings are essential for detecting and characterizing skeletal involvement [37,38]. The radiological characteristics of bone involvement related to SM are heterogeneous, including multiple focal sclerotic bone lesions, diffuse round sclerotic foci, and areas of normal or reduced bone density, primarily affecting the axial skeleton, ribs, humerus, and femur [35]. However, these lesions may resemble osteopoikilosis (a benign bone dysplasia characterized by numerous bone islands typically grouped around joints in metaepiphyseal regions, carpal and tarsal bones, pelvic ring, and scapulae) or metastases, complicating the diagnosis [39,40].

X-Ray and dual-energy X-Ray absorptiometry (DEXA) are crucial first-line imaging modalities for diagnosing and evaluating skeletal abnormalities due to their simplicity, low cost, and wide availability. While X-Rays are limited in detecting bone marrow changes and require significant bone loss to appreciate decreased bone density, DEXA is the gold standard for diagnosing osteoporosis and predicting fracture risk [41,42]. DEXA should be recommended for patients with idiopathic osteoporosis and symptoms related to mast cell mediator release, and for SM patients at diagnosis and during follow-up to identify those who may benefit from anti-osteoporotic treatment [43].

Meyer et al. found that DEXA results are positively correlated with serum tryptase levels and mast cell counts in bone marrow biopsies. Their study of 39 patients (18 women and 21 men) revealed significant differences in bone mineral density (BMD) between indolent and aggressive types of SM. They assessed the cKit mutation, serum tryptase levels, alkaline phosphatase, serum calcium levels, hemoglobin levels, leukocytes, and platelets, as well as bone marrow biopsies. In all patients, no significant differences were found between the different bone marrow patterns regarding cKit mutations. Correlation analysis showed an association between BMD and tryptase levels (r = 0.35, *p* = 0.049), the proportion of mast cells in the bone marrow biopsy (r = 0.45, *p* = 0.01), and years since diagnosis (r = −0.42, *p* = 0.02). These results confirm the utility of DEXA in SM beyond just the evaluation of osteopenia and osteoporosis [10].

In a study of 61 patients with SM, Riffel et al. correlated the prevalence of osteoporosis, increased BMD, and osteosclerosis with clinical parameters, disease type, and prognosis. They found that increased BMD and osteosclerosis are common in advanced SM but not in indolent SM. Advanced SM with elevated BMD and osteosclerosis was associated with a more aggressive phenotype, high-risk molecular aberrations, and poorer survival. Osteoporosis was detected in 38% of ISM patients but only in 6% of advanced SM patients (*p* = 0.004). An increase in BMD was found in 3% of ISM patients and in 75% of advanced SM patients (*p* < 0.001), while osteosclerosis was observed only in advanced SM patients (50%). Patients with AdvSM who exhibited increased BMD had higher levels of mast cell infiltration in the bone marrow, as well as elevated serum tryptase and alkaline phosphatase levels compared to patients with indolent systemic mastocytosis. Furthermore, these AdvSM patients had a greater number of high-risk molecular mutations (*p* < 0.05). The prognosis for AdvSM patients with increased BMD was worse than for those without increased BMD, with a median overall survival of 3.6 years compared to no survival (*p* = 0.031) [11].

It is important to evaluate laboratory tests such as serum levels of 25-OH vitamin D, s-CTX (C-terminal telopeptide of type I collagen), parathyroid hormone, alkaline phosphatase, calcium, phosphorus, 24-h urinary calcium, and phosphaturia [44]. Carosi et al. demonstrated a correlation between C-telopeptide and osteoprotegerin with serum tryptase levels and mast cell counts. However, due to the lack of high reliability in serum tryptase values, a bone marrow biopsy may be necessary when clinical suspicion is high. Hypertryptasemia is commonly observed in systemic mastocytosis. This study evaluated the prevalence of hypertryptasemia among patients with severe osteoporosis, assessed the efficacy of the tryptase test for diagnosing SM in these individuals, and examined their bone characteristics. The study reviewed clinical records of 232 patients (168 women and 64 men) diagnosed with osteoporosis, of whom 50.4% had fractures and had undergone serum tryptase testing. Bone marrow evaluations were conducted in a subgroup of patients with hypertryptasemia, and clinical, biochemical, and radiographic data were collected. Hypertryptasemia was identified in 33 of these patients.

Bone marrow evaluations were conducted on 16 patients with hypertryptasemia. Of these, 8 had normal bone marrow results, 3 met the criteria for a diagnosis of systemic mastocytosis, 4 showed mast cell abnormalities, and 1 had polycythemia vera. All patients with bone marrow abnormalities had serum tryptase levels greater than 11.4 ng/mL. The optimal cut-off for serum tryptase levels related to bone marrow abnormalities was found to be 17.9 ng/mL, with a sensitivity of 75% (AUC = 0.797, *p* = 0.015 according to ROC analysis). Every osteoporotic patient with hypertryptasemia had at least one vertebral fracture and experienced a significant reduction in lumbar bone mineral density. Mast cell-related disorders accounted for 3.0% of cases of severe osteoporosis and 7.4% of secondary causes of osteoporosis, suggesting that these disorders may contribute to bone fragility. Therefore, the assessment of serum tryptase remains valuable for identifying mast cell-related disorders [12].

## 6. Therapeutic Approach

From a therapeutic standpoint, there are various treatment options depending on the patient’s clinical picture. Antiresorptive medications are the cornerstone of treatment for osteoporosis induced by mastocytosis [45]. Brumnsen et al. demonstrated that bisphosphonate therapy allows for long-term bone protection despite persistent mast cell activity in a patient with osteoporosis and bone marrow mastocytosis [46]. However, patients with fractures prior to bisphosphonate therapy have a high risk of sustaining new fractures, as shown by Onnes et al. In this study, the long-term effects of bisphosphonate treatment on fractures, bone mineral density (BMD), and bone resorption were evaluated in patients with ISM in daily clinical practice. Retrospective analysis was conducted on ISM patients who received bisphosphonates due to osteoporosis and/or fractures (n = 58). Fractures were recorded using vertebral fracture assessment, thoracolumbar X-Rays, medical records, and a questionnaire. The five-year analysis (n = 30) was performed by comparing the observed five-year fracture risk with the predicted fracture risk from MastFx for untreated ISM patients and analyzing the five-year change in BMD and serum C-telopeptide (sCTx).

During a mean follow-up of 7.3 years, 14 of the 58 patients experienced 40 fractures. The five- and ten-year fracture-free survival rates were 81.9% (standard error [SE], 5.5%) and 67.0% (SE, 7.7%), respectively. The risk of fractures was significantly higher in patients with a previous vertebral fracture (*p* = 0.004), baseline femoral BMD lower than normal (*p* = 0.042), and a history of anaphylaxis (*p* = 0.028). No reduction in fracture risk was demonstrated over a five-year period, likely due to the small sample size. The Z-score for lumbar BMD significantly increased from a median (interquartile range [IQR]) of −2.20 (from −2.80 to −1.50) to −1.50 (from −2.30 to −0.60) (*p* < 0.001, n = 27). The Z-score for sCTx decreased from a median of 0.71 (IQR, −0.59 to 2.39) to −0.95 (−1.30 to −0.16) (*p* = 0.008, n = 15). In conclusion, bisphosphonates significantly increase BMD and decrease sCTx in patients with ISM. However, fractures occur frequently, especially in patients with a history of previous fractures [13].

In particular, Rossini et al. [14] observed that 5 mg of zoledronic acid IV annually could be an effective treatment option to reduce bone turnover markers and prevent bone loss. Unfortunately, a common adverse effect, compared to the general population, is the acute phase reaction to the first administration of zoledronic acid; this is a transient reaction and can also be managed with premedication and providing adequate information to the patient. Twenty-five patients with osteoporosis secondary to indolent systemic mastocytosis received a single intravenous infusion of 5 mg of zoledronic acid dissolved in 100 mL of 0.9% saline over 60 min. After one year, the average increase in BMD was 6.0% ± 4.4% at the spinal level and 2.4% ± 3.2% at the total hip level. Serum levels of bone turnover markers decreased from baseline: bone alkaline phosphatase by −34% and −35%, and C-terminal telopeptide by −68% and −56% at 6 and 12 months, respectively. None of the patients reported new fractures during the one-year follow-up. A transient acute phase reaction was observed in all of the first 20 treated patients, but this was prevented in 4 of the subsequent 5 patients who received regular paracetamol during the three days following the infusion.

Therefore, a single intravenous infusion of 5 mg of zoledronic acid in patients with osteoporosis secondary to indolent systemic mastocytosis is associated with significant increases in bone mineral density (BMD) of the spine and hip, as well as decreases in bone turnover markers for at least one year. Another therapeutic option, when the use of bisphosphonates is contraindicated, is Denosumab, considering the central role that the RANK-RANKL system seems to play in the pathogenesis of osteoporosis caused by SM. The use of teriparatide, the active fragment 1–34 of parathyroid hormone, is not recommended as it may further promote the growth and proliferation of abnormal mast cells and induce more aggressive forms of SM, as analyzed by Rossini et al. [47]. In fact, denosumab appears to be a valid alternative for treating bone loss even in ISM patients intolerant to bisphosphonates, where the risk of side effects and new fractures is minimal, as observed by Orsolini et al. The purpose of this study was to investigate the therapeutic effect of denosumab, an anti-RANKL monoclonal antibody, for the treatment of bone loss in patients with indolent systemic mastocytosis intolerant to bisphosphonates. Four patients gave informed consent for treatment with 60 mg of denosumab administered subcutaneously every six months, following the same regimen used for postmenopausal osteoporosis. Bone mineral density (BMD) was measured at lumbar and femoral sites at baseline and after one year. The C-terminal telopeptide of type I collagen (CTX), bone alkaline phosphatase, and serum tryptase levels were determined at baseline and after 12 months with fasting blood samples. BMD significantly increased at both sites over 12 months; all patients showed a marked decrease in serum CTX levels and a smaller decrease in serum bALP levels. After treatment with denosumab, a decrease in serum tryptase levels was observed in all patients, and no adverse events or new fractures occurred [15].

Cytoreductive drugs are currently indicated only in advanced/aggressive forms of SM, but they may also be considered for patients with severe ISM osteoporosis who experience new fractures despite treatment or in patients refractory to bisphosphonate therapy, after analyzing the risk-benefit ratio. IFN-α (interferon alpha) is considered the first-line cytoreductive agent. Weide et al. [16] observed a marked reduction in mast cell numbers in the bone marrow and a significant increase in mineralization and bone density in patients treated with IFNα-2b. Three patients with systemic mastocytosis and osteopenia were successfully treated, two of whom had pigmented urticaria and two experienced severe back pain due to vertebral compression fractures. All patients received a daily dose of interferon of 3 × 5 million units/week subcutaneously for a period of 6 months.

The therapy was well tolerated and the back pain resolved in both patients. Since IFN therapy is often discontinued or rescheduled due to the occurrence of flu-like reactions post-dose, Laroche et al. [17] reported that the combination of IFN and pamidronate resulted in a greater increase in bone marrow density in osteoporosis with concomitant mastocytosis. In four patients (mean age 52 years) with severe osteoporosis and mastocytosis confirmed by bone marrow biopsy (over 40 mast cells/mm^3^), the following protocol was applied: interferon alpha (IFN) 3 million units (MU) three times a week, reduced to 1.5 MU three times a week in case of intolerance, and pamidronate (Pam) 90 mg/month as an infusion. This treatment was administered for 2 years, followed by Pam alone at a dose of 90 mg/month. After 3–4 years of treatment, no patient presented new vertebral or non-vertebral fractures. The average increase in bone mineral density (BMD) with IFN and Pam was 16.05 ± 6.12% at the spinal level, 5 ± 2.24% at the femoral neck, and 4.12 ± 3.03% for the whole body; the increase or loss of bone mineral density with Pam alone was +0.2 ± 2.13% at the spinal level, −2.25 ± 2.78% at the femoral neck, and −0.1 ± 3.35% for the whole body. In one patient, the IFN dose was halved due to a flu-like syndrome, and in another, the IFN dose was interrupted after a year for the same reason. This increase was then maintained with monthly Pam infusions. The increase in bone density also reduces the risk of new vertebral and non-vertebral fractures, as further emphasized by Laroche et al. Ten patients with a mean age of 52.5 years with systemic mastocytosis and osteoporotic fractures were recruited for their study; these patients were treated with interferon alpha 1.5 million U three times a week in combination with monthly infusions of pamidronate (1 mg/kg) for 2 years, followed by pamidronate infusions every 3 months. Before treatment, the average number of vertebral fractures was 3.5, the spinal T-score was −3 ± 1, the hip T-score was −1.9 ± 0.7, the serum C-terminal telopeptide was 357 ± 258 pg/mL (N = 80–800), the bone alkaline phosphatase was 20 ± 3.2 UI (N = 8–25), and the tryptase was 49 ± 36 μg/mL (N < 10). Interferon alpha was discontinued in 2 patients due to poor tolerance. The mean follow-up was 60 months. No patient developed a fracture during treatment. In the 8 patients treated with interferon alpha and pamidronate, the mean annual increase in spinal bone mineral density was 12.6% ± 5.6% and in hip bone mineral density was 1.93%. The serum C-terminal telopeptide decreased by 66%, bone alkaline phosphatase decreased by 25%, and tryptase decreased by 34%. In the 2 patients treated with only pamidronate, the mean annual increase in bone mineral density was 2.4% ± 0.1% at the spinal level and 0% ± 0.1% at the hip. The combined treatment with low doses of interferon and pamidronate significantly increased bone density [18]. Early establishment of therapy with anti-mediators or agents acting on bone formation, such as the Wnt pathway targeting DKK-1 (Dickkopf-1) or sclerostin (SOST), for example, may reverse some bone changes related to the disease; however, there are currently no definitive studies on this. Rossini et al. [32] observed that serum levels of DKK1, but not of SOST, significantly increased in ISM patients with varying degrees of bone involvement and were correlated with PTH markers and bone turnover. Tyrosine kinase inhibitors are a promising group of drugs indicated in aggressive forms of SM as they target KIT; however, there are currently no data on their effects on BTM, BMD, and fracture risk. We must also consider that prescribing vitamin D and calcium supplements remains essential in all conditions ranging from mild osteopenia to severe osteoporosis, as emphasized by Zanotti et al. [44]. Finally, regarding a surgical approach to vertebral fractures due to osteoporosis secondary to mastocytosis, the most commonly used technique is kyphoplasty; in these patients, however, it should be conducted with appropriate precautions due to the potential risk of pressure-induced release of the mediator histamine and the resulting effects on the body, as observed by Kruger et al. [48].

## 7. Conclusions

The identification of bone involvement in patients with systemic mastocytosis is crucial due to the heterogeneity of manifestations and intervention strategies. An early diagnosis of osteoporosis secondary to mastocytosis allows for the initiation of appropriate pharmacological treatment. The literature demonstrates that there are several effective therapeutic options, which can lead to an increase in bone mineral density (BMD) and a reduction in fracture risk, thereby improving the patient’s quality of life. Finally, since this is a rare disease that requires a multidisciplinary approach, it is essential for patients to be followed in specialized centers for mastocytosis to receive comprehensive and coordinated care. This would ensure not only targeted treatment but also adequate monitoring and access to specific resources for disease management.

## Figures and Tables

**Table 1:** Search strategy.

“mastocytosis” [mesh] OR “osteoporosis” [mesh] OR “biphosfonates” [mesh] OR
“Denosumab” [mesh] OR “IFN-alfa” [mesh] OR “Teriparatide” [mesh] OR
“kyphoplasty” [mesh] OR “tyrosine kinase inhibitors” [mesh] OR “Dexa” [mesh] OR
“fracture risk” [mesh] OR osteosclerosis [mesh] OR “zoledronate” [mesh] OR “serume trypsin” [mesh] OR “bone lesions, focal or multiple” [mesh] OR “osteopenia” [mesh] OR “bone marrow biopsy” [mesh] OR “mast cells” [mesh] OR “bone scintigraphy” [mesh]

**Table 2:** Eligibility criteria.

**Inclusion Criteria**
English or French language
Reference period
Osteoporosis
Clinical, observational and retrospective studies, reviews
Studies that included drug therapies for osteoporosis in systemic mastocytosis
**Exclusion Criteria**
Books, case reports
Reference period
Focal or multiple bone lesions, MS sarcoma, osteosclerosis
Absence of bone involvement in SM
Other languages

**Table 3:** Characteristics and results of included studies.

Author, year	Title	Study Design	Total Group	Main Findings
Meyer et al., 2021 [10]	Bone mineral density in patients with systemic mastocytosis: correlations with clinical and histopathological features	Clinical observational	n°39 patients (18 women and 21 men)	DXA findings are associated with clinical and bone marrow biopsy parameters in SM. Has been identified a positive association with tryptase level and mast cell amount in bone marrow biopsies.
Riffel et al., 2020 [11]	An increased bone mineral density is an adverse prognostic factor in patients with systemic mastocytosis.	Clinical observational	n°61 patients with mastocytosis (ISM, n = 29, 48%; AdvSM, n = 32, 52%)	AdvSM patients with increased bone mineral density had higher levels of bone marrow mast cell infiltration, higher levels of serum tryptase and alkaline phosphatase than ISM patients, and a higher number of high molecular risk mutations. The prognosis of AdvSM patients with increased BMD is inferior to those without increased BMD
Carosi et al., 2020 [12]	Hypertryptasemia and Mast Cell-Related Disorders in Severe Osteoporotic Patients	Clinical Observational	No. 232 patients (168 females and 64 males)	There is a correlation of C-telopeptide and osteoprotegerin with serum tryptase levels and mast cell count, however, as serum tryptase values lack high reliability, when high clinical suspicion is present, a bone marrow biopsy may be necessary
Onnes et al., 2020 [13]	Fracture Risk Reduction by Bisphosphonates in Mastocytosis?	Clinical Observational Retrospective	n°58 patients	Bisphosphonates significantly increase BMD and decrease sCTx in patients with ISM. However, FFx still occurs frequently, especially patients with previous FFx
Rossini et al., 2014 [14]	Zoledronic acid in osteoporosis secondary to mastocytosis.	Clinical Observational	n°20 patients	A single intravenous infusion of 5 mg zoledronic acid in patients with osteoporosis secondary to indolent systemic mastocytosis is associated with significant increases in bone mineral density of the spine and hip and decreases in bone turnover markers over at least 1 year.
Orsolini et al., 2017 [15]	Denosumab for the Treatment of Mastocytosis-Related Osteoporosis: A Case Series	Clinical Observational	n°4 patients	BMD increased significantly during the 12 months of treatment; all patients had a major decrease in serum CTX levels and a minor decrease in serum bALP levels. After treatment with denosumab, a decrease in serum tryptase levels was also observed in all patients and no adverse events or new fractures occurred.
Weid et al., 1996 [16]	Successful treatment of osteoporosis in systemic mastocytosis with interferon alpha-2b	Clinical Observational	n°3 patients	In patients treated with IFNα-2b, a marked decrease in the number of mast cells in the bone marrow and a significant increase in mineralisation and bone density were observed.
Laroche et al., 2007 [17]	Clinical and densitometric efficacy of the association of interferon alpha and pamidronate in the treatment of osteoporosis in patients with systemic mastocytosis	Clinical Observational	n°4 patients	The association between IFN and pamidronate led to a greater increase in bone marrow density in osteoporosis with concomitant mastocytosis.
Laroche et al., 2009 [18]	Interferon alpha and pamidronate in osteoporosis with fracture secondary to mastocytosis	Clinical Observational	n°10 patients	Combined treatment with low doses of interferon and pamidronate significantly increased bone density.

## Data Availability

All data supporting the findings of this study are available within the paper and in references.

## Abbreviations

MS	Mastocytosis
TEP	Technical Expert Panel
PRISMA-ScR	Preferred Reporting Items for Systematic Reviews and Meta-Analyses Extension for Scoping Reviews
IFN-alfa	Interferone alfa
SM	systemic mastocytosis
SCF	stem cell factor
ISM	Indolent sistemic mastocytosis
KIT	gene encoding tyrosine kinase protein
BMD	Bone mineral density
SM-AHNMD	systemic mastocytosis with associated clonal non-mast cell hematologic disease
MC	mast cell
NSAIDs	non-steroidal anti-inflammatory drugs
DEXA	dual-energy X-Ray absorptiometry
DKK-1	Dickkopf-1
SOST	sclerostin
BTM	bone turnover marker
PAM	pamidronate
CTX	C-terminal collagen type I telopeptide
bALP	bone alkaline phosphatase
FFx	fractures
BMM	bone marrow mastocytosis
RANKL	nuclear factor kappa-B ligand
PPARγ	peroxisome proliferator-activated receptor gamma
AdvSM	Advanced sistemi mastocytosis
TGF-beta	tumor growth factor-beta
VEGF	vascular endothelial growth factor
FGF	Fibroblast growth factor
WNT	wingless-related integration site
